# Evolving spectrum of arrhythmogenic cardiomyopathy: Implications for Sports Cardiology

**DOI:** 10.1002/clc.24069

**Published:** 2023-06-25

**Authors:** Francesca Graziano, Alberto Cipriani, Dorottya Balla, Sergei Bondarev, Martina Perazzolo Marra, Barbara Bauce, Hajnalka Vágó, Domenico Corrado, Alessandro Zorzi

**Affiliations:** ^1^ Department of Cardiac, Thoracic and Vascular Sciences and Public Health University of Padua Padova Italy; ^2^ Department of Sports Medicine Semmelweis University Budapest Hungary; ^3^ Heart and Vascular Center Semmelweis University Budapest Hungary

**Keywords:** arrhythmogenic cardiomyopathy, athletes, preparticipation screening, sports cardiology, sudden cardiac death

## Abstract

Arrhythmogenic cardiomyopathy (ACM) is a genetic heart muscle disease, structurally characterized by progressive fibro‐fatty replacement of the normal myocardium and clinically by ventricular arrhythmias (VAs). Predominantly thanks to the use of cardiac magnetic resonance, we have learnt that the spectrum of the disease encompasses not only the classical right ventricular phenotype, but also biventricular and left dominant variants. Sport activity contributes to the phenotypic expression and progression of ACM and may trigger life‐threatening VAs and sudden cardiac death (SCD). We conducted a review of the literature about ACM and its implications in Sport Cardiology and summarized the main findings in this topic. Early identification of affected athletes through preparticipation screening (PPS) is fundamental but, while classical right‐ventricular or biventricular phenotypes are usually suspected because of electrocardiogram (ECG) and echocardiographic abnormalities, variants with predominant left ventricular involvement are often characterized by normal ECG and unremarkable echocardiography. Usually the only manifestations of such variants are exercise‐induced VAs and for this reason exercise testing may empower the diagnostic yield of the PPS. Patients with ACM are not eligible to competitive sports activity, but low‐to‐moderate intensity physical activity under medical supervision is possible in most cases.

## Graphical abstract

Preparticipation screening offers the possibility to early identify asymptomatic athletes with arrhythmogenic cardiomyopathy through step‐by‐step research for signs and subsequent further investigations. After the diagnosis, competitive sport activity should be discouraged and patients should be guided to safe recreational physical activity. CMR, cardiac magnetic resonance; ICD, implantable cardioverter defibrillator; PVBs, premature ventricular beats.

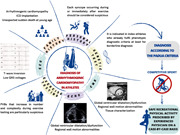

## 1 INTRODUCTION

**Figure 1:**
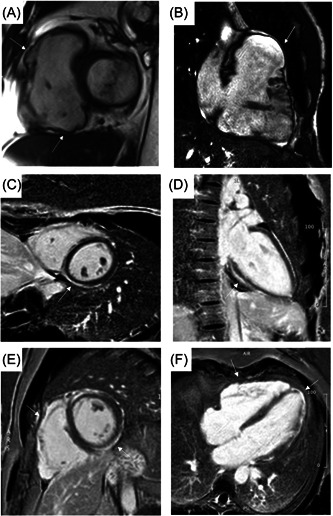
CMR images showing the three variants of arrhythmogenic cardiomyopathy. Cine image in short‐axis view (systolic frame) showing multiple sacculations of the RV walls (white arrows) of a right‐dominant variant of arrhythmogenic cardiomyopathy (A, B); adapted from Cipriani et al.^6^ Postcontrast phase in short axis (C) and 2‐chambers view (D) of a left‐dominant variant in a 23‐year‐old female patient with mutations in desmoplakin gene and in junction plakoglobin gene; adapted from Graziano et al.^7^ Postcontrast phase in short axis (E) and 4‐chambers view (F) of a biventricular variant in a 39‐year‐old male patient with desmocollin‐2 gene mutation; adapted from Graziano et al.^7^

Arrhythmogenic cardiomyopathy (ACM) is an inherited heart muscle disease characterized by the progressive loss of ventricular myocardium and its replacement with fibro‐fatty tissue.^1^ ACM is a genetically based disease; most commonly caused by mutations in genes encodings desmosomal proteins that mediate cell‐to‐cell adhesion. In a minority of patients the mutations affect nondesmosomal genes encoding ion channels and cytoskeletal components.^2^, ^3^ However, in just half of probands it is possible to demonstrate a genetic defect responsible for the disease and/or a positive family history.^4^ ACM is characterized by a wide phenotypic spectrum, which has recently broaden particularly thanks to the widespread use of tissue characterization by cardiac magnetic resonance (CMR): from the classical type with prevalent right ventricle (RV) involvement (Arrhythmogenic Right Ventricular Cardiomyopathy [ARVC]), to biventricular forms, which are the most common, and forms with predominant or exclusive left ventricular (LV) involvement (Arrhythmogenic LV Cardiomyopathy [ALVC])^5^ (Figure 1).

Ventricular electrical instability is typical of ACM, with a particular age‐related arrhythmic behavior due to its evolutive and progressive nature. Indeed, ventricular fibrillation (VF) and sudden cardiac death (SCD) are typical of younger patients, may be because of acute electrical instability in the context of the so‐called “hot‐phases,” which are characterized by myocarditis‐mediated bouts of acute myocyte necrosis. On the other hand, sustained monomorphic ventricular tachycardia (VT) is common in older patients and originates from re‐entry circuits around stable fibro‐fatty myocardial scars.^8^, ^9^ Ventricular arrhythmias (VAs) are typically triggered by sport activity and they are often the first clinical manifestation in asymptomatic subjects. Therefore, ACM is a leading cause of SCD in young and athletes.^10^, ^11^ Intense and prolonged physical exercise not only favor VAs but also promotes the phenotypic expression and progression of the disease.^12^, ^13^, ^14^

Preparticipation screening (PPS) offers the possibility to early identify asymptomatic patients with ACM and, by modulating the intensity and type of exercise, to reduce the risk of exercise‐related disease progression and SCD.^15^, ^16^, ^17^ However, while the classical RV or biventricular phenotype is usually suspected at PPS by resting electrocardiogram (ECG) abnormalities, variants with predominant LV involvement are often characterized by normal ECG and unremarkable echocardiography. Usually the only manifestations of such phenotypes are exercise‐induced VAs.

In this review we addressed the new diagnostic criteria of ACM that take into account the evolving spectrum of the disease, the diagnosis of the disease (both the classical and the LV variants) in the athlete and the implications for competitive sports eligibility.

## 2 THE PADUA CRITERIA

The diagnosis of ACM is challenging, because of the absence of a single sensitive and specific test. Therefore, formal criteria based on a multiparametric approach were introduced to facilitate and standardize the clinical diagnosis. In 1994 the first Task Force^18^ criteria were published, then revised in 2010.^19^ The lack of criteria for identifying LV involvement, the need to include late gadolinium enhancement (LGE) by CMR as well as the necessity to revise the existing RV criteria based on new scientific evidence were the basis for the creation of new criteria,^20^ the “Padua criteria,” which were published in 2020.

The “Padua criteria” are organized in two different sets of criteria to identify, respectively, clinical signs of RV and LV involvement. In both sets, the traditional organization in six diagnostic categories was maintained, which includes morpho‐functional changes, tissue characterization, repolarization and depolarization ECG abnormalities, VAs and family history/genetic testing. The criteria are differentiated in “major” and “minor” depending on their specificity for diagnosing ACM. Only one major or minor criterion for each category can be considered (Table 1).

**Table 1:** The Padua Criteria.

	Criteria for RV involvement	Criteria for LV involvement
**I. Morpho‐functional ventricular abnormalities**	*By 2D echocardiogram, CMR or angiography:*	*By 2D echocardiogram, CMR or angiography:*
	Major	Minor
	Regional RV akinesia, dyskinesia, or bulging plus one of the following:	Global LV systolic dysfunction (depression of LV EF or reduction of echocardiographic global longitudinal strain), with or without LV dilatation (increase of LV EDV according to the imaging test specific nomograms for age, sex, and BSA)
	‐ global RV dilatation (increase of RV EDV according to the imaging test specific nomograms for age, sex and BSA) ‐ global RV systolic dysfunction (reduction of RV EF according to the imaging test specific nomograms for age and sex)	
	Minor	Minor
	Regional RV akinesia, dyskinesia or aneurysm of RV free wall	Regional LV hypokinesia or akinesia of LV free wall, septum, or both
**II. Structural myocardial abnormalities**	*By CE‐CMR*:	*By CE‐CMR*:
	Major	Major
	Transmural LGE (stria pattern) of ≥1 RV region(s) (inlet, outlet, and apex in 2 orthogonal views)	LV LGE (stria pattern) of ≥1 Bull's Eye segment(s) (in 2 orthogonal views) of the free wall (subepicardial or midmyocardial), septum, or both (excluding septal junctional LGE)
	*By EMB (limited indications)*:	
	Major	
	Fibrous replacement of the myocardium in ≥1 sample, with or without fatty tissue	
**III. ECG repolarization abnormalities**	Major	Minor
	Inverted T waves in right precordial leads (V_1_, V_2_, and V_3_) or beyond in individuals with complete pubertal development (in the absence of complete RBBB)	Inverted T waves in left precordial leads (V_4_–V_6_) without complete LBBB
	Minor	
	‐ Inverted T waves in leads V1 and V2 in individuals with completed pubertal development (in the absence of complete RBBB) or ‐ Inverted T waves in V1, V2, V3 and V4 in individuals with completed pubertal development in the presence of complete RBBB	
**IV. ECG depolarization abnormalities**	Minor	Minor
	‐ Epsilon wave (reproducible low amplitude signals between end of QRS complex to onset of the T wave) in the right precordial leads (V1 to V3) or ‐ Terminal activation duration of QRS ≥55 ms measured from the nadir of the S wave to the end of the QRS, including R’, in V1, V2, or V3 (in the absence of complete RBBB)	Low QRS voltages (<0.5 mV peak to peak) in limb leads (in the absence of obesity, emphysema, or pericardial effusion)
**V. Ventricular arrhythmias**	Major	Minor
	Frequent ventricular extrasystoles (>500 per 24 h), non‐sustained or sustained ventricular tachycardia of LBBB non‐inferior axis morphology	Frequent ventricular extrasystoles (>500 per 24 h), non‐sustained or sustained ventricular tachycardia with a RBBB morphology (excluding the “fascicular pattern”)
	Minor	
	Frequent ventricular extrasystoles (>500 per 24 h), non‐sustained or sustained ventricular tachycardia of LBBB morphology with inferior axis (“RVOT pattern”)	
**VI. Family historygenetics**	Major	
	‐ ACM confirmed in a first‐degree relative who meets diagnostic criteria or ‐ ACM confirmed pathologically at autopsy or surgery in a first‐degree relative or ‐ Identification of a pathogenic or likely pathogenetic ACM mutation in the patient under evaluation	
	Minor	
	‐ History of ACM in a first‐degree relative in whom it is not possible or practical to determine whether the family member meets diagnostic criteria or ‐ Premature sudden death (<35 years of age) due to suspected ACM in a first‐degree relative or ‐ ACM confirmed pathologically or by diagnostic criteria in second‐degree relative	

*Note*: Adapted from Corrado et al.^21^

Abbreviations: ACM, arrhythmogenic cardiomyopathy; BSA, body surface area; CE‐CMR, constrast enhanced‐cardiac magnetic resonance; CMR, cardiac magnetic resonance; EDV, end diastolic volume; EF, ejection fraction; EMB, endomyocardial biopsy; LBBB, left bundle branch block; LGE, late gadolinium enhancement; LV, left ventricle; RBBB, right bundle branch block; RV, right ventricle; RVOT, right ventricular outflow tract.

An important element of novelty of the Padua Criteria is that, because ACM is a structural disease, at least one morpho‐functional or structural criterion needs to be satisfied. Another is that noninvasive tissue characterization by CMR and invasive endomyocardial biopsy were placed side‐by‐side.

**Figure 2:**
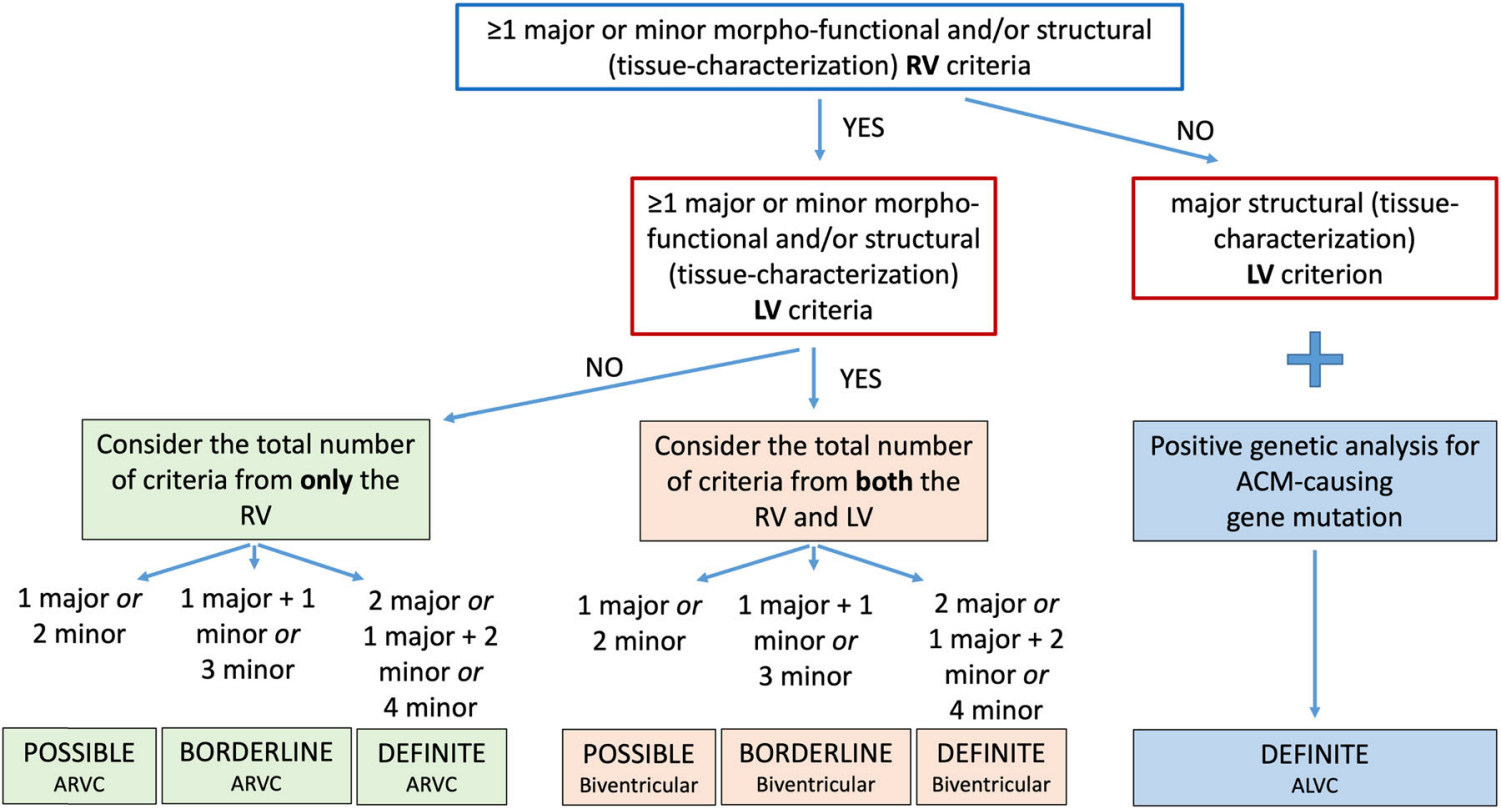
Flowchart for phenotypic characterization of arrhythmogenic cardiomyopathy. ACM, arrhythmogenic cardiomyopathy; ALVC, arrhythmogenic left ventricular cardiomyopathy; ARVC, arrhythmogenic right ventricular cardiomyopathy; LV, left ventricle; RV, right ventricle. Reproduced from Graziano et al.^4^

According to the number of positive LV and RV criteria, the diagnosis can be one of the three different phenotypic variants: “right‐dominant” variant, which is the classical form with RV involvement; “biventricular” variant, with involvement of both ventricles; “left‐dominant” variant, with only LV involvement. After that, the likelihood of disease is defined according to the combination of major and minor criteria that are fulfilled, and the diagnosis can be “definite,” “borderline,” or “possible” (Figure 2). The diagnosis of ALVC, which can only be “definite,” requires identification of a pathogenic or likely pathogenic ACM‐causing gene mutation. The rationale for the need of positive genotyping testing lays in the possible overlap between left‐dominant ACM and phenocopies such as dilated cardiomyopathy, cardiac sarcoidosis or myocarditis. Indeed, investigating the disease etiology and differentiating ACM from phenocopies is the last and challenging step to reach the diagnosis.^22^

## 3 CLASSICAL PHENOTYPE OF ACM: HOW TO SUSPECT AND DIAGNOSE IT IN ATHLETES

PPS is a fundamental tool for early diagnosing ACM in asymptomatic athletes (Table 2). Medical evaluation should include personal and family history, physical examination, and resting ECG.^15^ In addition, in Italy the use of exercise testing (ET) has empowered the diagnostic yield of PPS evaluation through the detection of premature ventricular beats (PVBs), which sometimes represent the only clinical manifestation of the disease.^23^, ^24^, ^25^, ^26^

**Table 2:** The warning signs that should raise the suspicion of arrhythmogenic cardiomyopathy in the athlete undergoing preparticipation screening.

Tool	Abnormality	Note
**Family history**	ACM	Family history should not be limited to first‐degree relatives because of incomplete penetrance of ACM.
	ICD implantation	
	Unexpected sudden death at young age	
**Personal history**	Syncope	Each syncope occurring during or immediately after exercise should be considered suspicious.
	Palpitations	
	Pericarditis‐like chest pain	Myocarditis‐like phases (“hot‐phases”) characterized by chest pain, ST segment elevation on the ECG and troponin rise may occur during the natural history of ACM.
**Physical examination**	Usually unremarkable	
**Resting 12‐leads ECG**	Low QRS voltages in the limb leads	
	T‐wave inversion	Comparison with previous ECG is useful to identify dynamic changes.
		T‐wave inversion in V1‐V2/V3 in prepubertal children are a normal finding.
		T‐wave inversion preceded by J‐point/ST segment elevation confined to V1‐V4 are common in healthy black athletes.
	Epsilon wave	Typical of the advanced stages of disease.
	RBBB	Classified as borderline finding in athletes, it is suspicious when associate with other signs of ACM.
	Premature ventricular beats	
**Exercise testing**	Premature ventricular beats	Analysis of the ectopic QRS morphology is helpful for risk stratification.
		Premature ventricular beats that increase in number and complexity during exercise testing are particularly suspicious.
		Normalization of negative T waves during exercise is not specific.
**Echocardiography**	RV wall motion abnormalities	Specific views for the complete assessment of the RV should be performed.
	LV involvement should be unremarkable	The cut‐off values used should be specific for athletes.
**Cardiac Magnetic Resonance**	RV function/wall motion abnormalities associated with transmural LGE	RV wall motion abnormalities associated with abnormal tissue characterization are more specific for RV involvement.
	LV LGE with a nonischemic pattern	The cut‐off values used should be specific for athletes.
**Genetic test**	Pathogenic or likely‐pathogenic ACM‐causing gene mutation	It is indicated in an index athlete who already fulfils phenotypic diagnostic criteria at least for borderline ACM.

Abbreviations: ACM, arrhythmogenic cardiomyopathy; ICD, implantable cardioverter defibrillator; LGE, late gadolinium enhancement; LV, left ventricle; RV, right ventricle; TWI, T wave inversion.

A *personal history* positive for pericarditis and/or myocarditis, especially if recurrent, should be thoroughly investigated in suspecting a “hot phase” of ACM.^27^, ^28^ Palpitations could be a sign of VAs, such as pre‐syncope and syncope. Indeed, fast and self‐limiting VT may cause transient loss of consciousness. Consequently, a history of syncope, especially occurring during or immediately after exercise and not clearly associated with specific circumstances, such as standing for long period or pain, should be carefully evaluated.^29^, ^30^ Because of the incomplete penetrance of the disease, *family history* should be extended beyond first‐degree relatives. Relevant information is unexplained sudden death at young age, ICD implantation, and of course familial ACM.

The *ECG* shows abnormalities in the majority of ARVC patients and in up to 90% of patients with a history of VT.^31^ The resting ECG could reveal a typical ARVC sign: T wave inversion (TWI) in V1−V3, which may extend to lateral and inferior leads depending on a more severe RV dilatation.^32^ T‐wave inversion preceded by J‐point and ST segment elevation may represent an early‐repolarization variant that is typical of athletes of Afro‐Caribbean descent.^33^ In young peripubertal athletes there may be doubts about the persistence of a juvenile repolarization pattern. In this case the comparison with a previous ECG is fundamental to understand if there is a trend toward normalization or TWI is a new pathological evidence.^23^, ^34^ Moreover, persistence of normalization of TWI during exercise testing is nonspecific and does not allow differential diagnosis between benign TWI and ACM.^35^ The epsilon wave is another typical sign of ARVC that can be observed in advanced stages of the disease, and it is usually found in association with other ECG and clinical abnormalities. Finally, complete right bundle branch block (RBBB) is considered a borderline abnormality in athlete's ECG, and it is typical of advanced ARVC stages. So, the presence of RBBB in isolation or with other recognized physiological electrical patterns of athletic training does not warrant further assessment in asymptomatic athletes without a family history suspicious for ACM.^36^ Otherwise, the combination of a complete RBBB with other signs of ACM in ECG, family and personal history and ET should be closely investigated.

The *ET* is essential in placing the suspicion of ACM. This is because VAs, sometimes even isolated PVBs, are often the first manifestation in both right‐ and left‐dominant forms. PVBs or VT with LBBB/superior axis morphology are more specific for ACM as they originate from the RV free wall or interventricular septum. Instead, VAs with a LBBB/inferior axis morphology are less specific (minor criterion), and are often idiopathic, in keeping with an origin from RV outflow tract, but they should still be investigated at least by echocardiography.^37^, ^38^ The traditional idea is that the risk of an underlying disease correlates with number of PVBs, and the International recommendations classify as abnormal the recording of at least two PVBs on a single ECG tracking in athletes.^36^ However, recent evidence suggests that morphology, complexity and relation with exercise are more accurate characteristics than the number in differentiating between probable benign and potentially malignant PVBs.^37^ In addition, the reproducibility of VAs at repeat ET in terms of morphology and exercise response is a useful tool for risk stratification in athletes presenting PVBs at PPS.^39^

In case of abnormal finding at PPS, second and third level investigations should be performed. The *24‐h ECG Holter* (possibly with 12 leads to allow evaluation of PVBs morphologies) including a training session is an important second‐level test not only to record the VA burden over a longer period, but also to assess the occurrence of arrhythmias during the habitual sports activity.

*Echocardiography* and *CMR* are prescribed respectively as second and third level imaging investigations in case ACM is suspected.^40^ The physiological remodeling of the athlete's heart is characterized by an increase in RV volume that goes beyond the upper limits reported for the general population, so the cut‐off values used should be specific for athletes.^41^, ^42^ Moreover, the diagnostic criteria for ACM require the combination of RV dilatation or dysfunction with regional wall motion abnormalities, which are due to the presence of a fibro‐fatty scar and are not typical of the athlete's heart. It must be taken into account that the infero‐basal (sub‐tricuspid) RV region is a common site of wall motion abnormalities in ACM: this area is not evaluated by standard echocardiographic views and, for this reason, when ACM is suspected an off‐axis 2‐chamber apical view focused on evaluation of the inferior RV wall should be obtained.

In comparison to echocardiography, CMR allows a more precise assessment of RV function and provides tissue characterization.^6^, ^43^ The contribution of RV LGE technique to the diagnosis of ACM is particularly high when combined with wall motion abnormalities in the same area, because akinesia or dyskinesia in ACM are the results of myocardial scarring. On the other hand, evaluation of RV LGE may be challenging because of the thin RV wall (limiting sensitivity) and of artifacts such as slowly flowing or stagnant blood in the RV trabeculae causing LGE‐mimicking (limiting specificity). On the other hand, up to 70% of patients with RV disease also shows LV involvement in the form of subepicardial LGE: the combination of RV morpho‐functional abnormalities and LV LGE with a nonischemic distribution supports the diagnosis of biventricular ACM.

*Molecular genetic testing* is indicated in an index athlete who already fulfils phenotypic diagnostic criteria at least for borderline ACM, while it should not be used in the absence of a clear clinical suspicion (e.g., in athletes with PVBs or TWI and no structural abnormalities) because the results of genetic testing are often not clear‐cut and may be confounding.^44^, ^45^

## 4 THE CHALLENGING DIAGNOSIS OF LV VARIANTS

The incidence of SCD due to ARCV has been significantly reduced thanks to the routine use of ECG during PPS, leaving left‐dominant variants as an emergent substrate at post‐mortem investigation.^11^ The obstacle in suspecting and diagnosing ALVC can be traced to the fact that traditional investigations, such as ECG and echocardiography, have low sensibility if compared with the classical phenotype.

The *ECG* in ALVC is often normal. The presence of TWI in V4‐V6 is considered an abnormal finding in an athlete's ECG, and it is a criterion for ALVC (minor, because it is non‐disease specific). The physiological ventricular remodeling in athletes is characterized by increase in QRS voltages. Therefore, the presence of low‐amplitude QRS complexes (peak to peak < 0.5 mV) in the limb leads should be considered suspicious even if it is not included in the current recommendations for interpretation of the athlete's ECG, because it can reflect the decrease of LV myocardial mass by fibro‐fatty replacement and requires additional examination to be performed.^25^ This ECG abnormalities represent a risk factor for VAs and SCD in ACM patients.^31^

In ALVC, the *echocardiography* is often normal because the wave‐front of myocardial loss and fibrofatty replacement in the LV wall proceeds from the epicardium to the endocardium, typically in the inferior‐lateral wall, sparing the subendocardial layer which mostly accounts for the regional LV wall contractility.^6^

**Figure 3:**
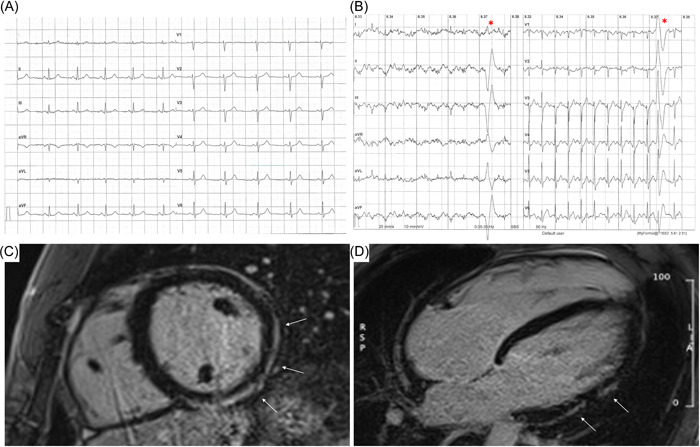
A 26‐year‐old competitive hockey player presented with a normal ECG (A), and frequent PVBs with right bundle branch block/superior axis morphology at high workload during exercise testing (B, red asterisks). The echocardiography was normal but post‐contrast sequences on CMR revealed a subepicardial stria of LGE with a “ring‐like” pattern, involving the anterior, lateral and inferior LV walls in their basal and medium portions (white arrows; C, short‐axis view; D, 4‐chamber view). As genetic testing was positive for desmoplakin gene mutation a diagnosis of ALVC was made. Adapted from Brunetti et al.^38^

Therefore, as mentioned above, VAs with a RBBB morphology/QRS > 130 msec are often the only manifestation of ALVC, so *ET* and any subsequent *Holter ECG* (preferably 12‐lead and including high‐intensity training session) are key examinations for raising the suspicion of ALVC (Figure 3). It should be noted that PVBs are typically adrenergic‐dependent but do not necessarily persist until the (or only appear at) peak of ET.^24^, ^39^ In case of high‐risk VAs features, *CMR* should be prescribed to athletes to rule out ALVC or other forms of nonischemic myocardial scarring even when echocardiography and ECG are normal.^37^, ^38^

## 5 MANAGEMENT OF ATHLETES WITH ACM

**Figure 4:**
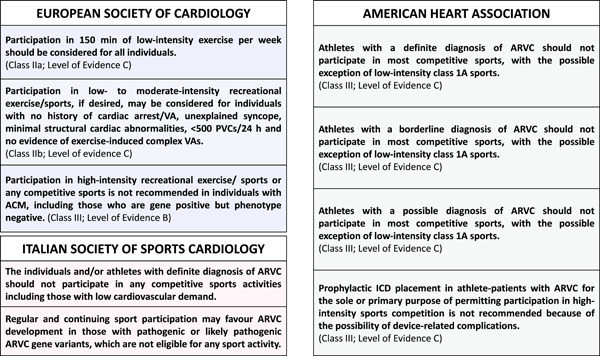
Recommendations of the European Society of Cardiology, American Heart Association, and Italian Society of Sports Cardiology in individuals with arrhythmogenic cardiomyopathy.^46^, ^47^, ^48^ ACM, arrhythmogenic cardiomyopathy; ARVC, arrhythmogenic right ventricular cardiomyopathy; ICD, implantable cardioverter defibrillator; PVCs, premature ventricular contractions; VAs, ventricular arrhythmias.

Figure 4 summarizes the recommendations for competitive sports participation and leisure‐time physical activity according to recent guidelines, which agree that competitive sport activity should be discouraged in athletes with ACM. The evidence is based on retrospective studies including patients with the classical phenotype of ARVC, which demonstrated that competitive sports activity may: (1) promote the development of disease in subjects with positive genotype and negative phenotype^13^; (2) worsen the degree of RV dysfunction in subjects with overt disease; (3) facilitate VAs (including appropriate ICD interventions^49^). Indeed, intense physical practice seems to be associated with a fivefold increase in the risk of SCD when compared to sedentary patients.^16^ As far as ALVC is concerned, there is solid evidence about the increased arrhythmic risk induced by competitive sport,^39^ but we currently do not know whether physical activity promotes disease penetrance and phenotype progression. At present, guidelines do not make distinctions between phenotypic variants.

The fact that engaging in competitive sports activity may be dangerous does not mean that any exercise should be avoided. Young subjects diagnosed with ACM should be guided to safe recreational physical activity prescribed by experienced physician on a case‐by‐case basis and avoid a sedentary behavior that could carry adverse consequences (e.g., depression, obesity, increased risk of coronary artery, social deprivation). Lie et al. evaluated the relative effects of exercise and intensity, and their relationship to disease progression in ARVC patients and family members with positive genotype. They observed more prevalent VAs in patients performing exercise of greater intensity (74% vs. 20% performed > 6 METS, *p* < .001) and volume (65%, vs. 30% exercised > 2.5 h/week, *p* < .001). Moreover, at multivariate analysis intensity rather than duration was independently associated with arrhythmias.^50^ The outcome of ACM patients involved in low‐intensity exercise was no worse than sedentary subjects, not increasing the risk of SCD, but benefiting of leisure time exercise.^51^ According to the European Society of Cardiology guidelines on sports cardiology, 150 min of low‐moderate intensity exercise should be recommended in all subjects with ACM with individualized prescription.

## 6 CONCLUSIONS

The early diagnosis of ACM through PPS in competitive athletes may reduce the disease progression and the risk of SCD, modifying its natural history. Sometimes the diagnosis of ACM can be challenging because of the potential overlap with the athlete's heart and the low sensitivity of resting ECG and echocardiography in ALVC. ET can empower the diagnostic yield of the PPS with the detection of exercise‐induced PVBs that are often the only manifestation of the disease. In the athlete, the diagnosis of ALVC relies on the tissue characterization ability of CMR, which should be performed in selected cases with high‐risk VAs irrespective of ECG and echocardiography findings. Finally, although competitive sports may be dangerous in patients with ACM, prescribing adequate and safe exercise by experienced physicians should prevent the diagnosis of ACM from becoming a reason for sedentariness that can be equally harmful for patients.

## CONFLICT OF INTEREST STATEMENT

The authors declare no conflict of interest.

## Data Availability

Data sharing is not applicable to this article as no new data were created or analyzed in this study.
